# Morpho-functional analysis of patient-derived xenografts reveals differential impact of gastric cancer and chemotherapy on the tumor ecosystem, affecting immune check point, metabolism, and sarcopenia

**DOI:** 10.1007/s10120-022-01359-w

**Published:** 2022-12-19

**Authors:** A. Venkatasamy, E. Guerin, W. Reichardt, V. Devignot, M. P. Chenard, L. Miguet, B. Romain, A. C. Jung, I. Gross, C. Gaiddon, G. Mellitzer

**Affiliations:** 1 Streinth Lab (Stress Response and Innovative Therapies), Inserm UMR_S 1113 IRFAC, Interface Recherche Fondamental et Appliquée à la Cancérologie, 3 Avenue Molière, 67200 Strasbourg, France; 2 IHU-Strasbourg, Institute of Image-Guided Surgery, 67200 Strasbourg, France; 3 Medizin Physik, Universitätsklinikum Freiburg, Kilianstr. 5a, 70106 Freiburg, Germany; 4 Pathology Department, Hôpital de Hautepierre, Hôpitaux Universitaires de Strasbourg, 1 Avenue Molière, 67098 Strasbourg Cedex, France; 5 Digestive Surgery Department, Hôpital de Hautepierre, Hôpitaux Universitaires de Strasbourg, 1 Avenue Molière, 67098 Strasbourg Cedex, France; 6 Laboratoire de Biologie Tumorale, Institut de Cancérologie Strasbourg Europe, 67200 Strasbourg, France

**Keywords:** Gastric cancer, Cisplatin, Magnetic resonance imaging, Mouse models, PD-L1, Immune checkpoint signaling hypoxia, p53

## Abstract

**Objectives:**

Gastric cancer (GC) is an aggressive disease due to late diagnosis resulting from the lack of easy diagnostic tools, resistances toward immunotherapy (due to low PD-L1 expression), or chemotherapies (due to p53 mutations), and comorbidity factors, notably muscle atrophy. To improve our understanding of this complex pathology, we established patient-derived xenograft (PDX) models and characterized the tumor ecosystem using a morpho-functional approach combining high-resolution imaging with molecular analyses, regarding the expression of relevant therapeutic biomarkers and the presence of muscle atrophy.

**Materials and methods:**

GC tissues samples were implanted in nude mice. Established PDX, treated with cisplatin or not, were imaged by magnetic resonance imaging (MRI) and analyzed for the expression of relevant biomarkers (p53, PD-L1, PD-1, HER-2, CDX2, CAIX, CD31, a-SAM) and by transcriptomics.

**Results:**

Three well-differentiated, one moderately and one poorly differentiated adenocarcinomas were established. All retained the architectural and histological features of their primary tumors. MRI allowed in-real-time evaluation of differences between PDX, in terms of substructure, post-therapeutic changes, and muscle atrophy. Immunohistochemistry showed differential expression of p53, HER-2, CDX2, a-SAM, PD-L1, PD-1, CAIX, and CD31 between models and upon cisplatin treatment. Transcriptomics revealed treatment-induced hypoxia and metabolic reprograming in the tumor microenvironment.

**Conclusion:**

Our PDX models are representative for the heterogeneity and complexity of human tumors, with differences in structure, histology, muscle atrophy, and the different biomarkers making them valuable for the analyses of the impact of platinum drugs or new therapies on the tumor and its microenvironment.

**Supplementary Information:**

The online version contains supplementary material available at 10.1007/s10120-022-01359-w.

## Introduction:

Third cancer worldwide in terms of mortality, gastric cancer (GC) has a very poor prognosis, with a 5-year overall survival < 25% [1, 2]. The reasons for this dramatic clinical situation are multiple but the lack of acceptable and affordable diagnostic tools for early detection and limited treatment options is the main one. Recently, the Cancer Genome Atlas (TGCA) research network published a classification of GC according to their molecular characteristics correlating with distinct clinical and histological characteristics [3, 4]. However, so far, this classification has not brought any concrete novel input in the clinical routine, neither by defining clear markers for therapeutic response, nor novel drugs for actionable therapeutic targets [5]. So far, surgical gastrectomy combined with platinum-based compounds (e.g., oxaliplatin) perioperative chemotherapy is the cornerstone of current treatments but outcome remains unfavorable, with a 30–40% response rate and a median survival < 1 year in advanced or metastatic stages**.** Furthermore, chemotherapy side effects (e.g., nephrotoxicity, gastro-intestinal toxicity, or neurotoxicity) [6, 7], together with de novo or acquired resistances (up to 75%), limit its use and effectiveness*.* Although immunotherapy, using immune check point inhibitors like pembrolizumab targeting PD-1, has been a revolution for the treatment of various cancers, including metastatic melanoma, favorable results in gastric cancer were only achieved for a small subset of tumors [8–11]. In particular, the expression of PD-L1 in the predominant “intestinal subtype” of GC (45%), which largely matches the “chromosome instability (CIN) molecular subgroup”, is relatively low and displays a poor response to immunotherapy, similarly to the “genetic stable subgroup” that is more frequent in younger patients and displays a poorer prognosis [3, 5]. Hence, the improvement of immunotherapy response requires a better understanding of the expression and function of PD-L1 in GC and its consequences on the immune landscape, within the tumor microenvironment. Similarly, the scientific view of cancers is shifting from organ-centered diseases to a more global and systemic approach. For instance, in gastric cancer, around 30–60% of GC patients develop cancer cachexia, presenting a progressive loss of adipose tissue and skeletal muscle mass [12, 13], which are enhanced upon chemotherapy or immunotherapy [13–15]. However, the respective impact of the gastric tumor itself and the treatment on muscle atrophy has not been much addressed so far, mainly due to the lack of adequate animal models. In this respect, for preclinical studies, patient-derived xenografts (PDX) mouse models are valuable tools in cancer research, especially for anti-cancer drug testing in preclinical studies. PDX tumors remain stable across generations and retain the major traits of the primary tumor, thus resulting in the only model able to reflect the vast patient and tumor tissue heterogeneity. PDX allow repeated measures and time-course follow-up of tumor progression, in response to various conditions (e.g., anti-cancer drugs, genetic modifications…), as well as an analysis of the evolution of intra-tumoral clonal variation [16–21].

In this study, we aimed to establish novel gastric cancer PDX models (GCX) that will allow us to reproduce the intra- and inter-tumoral clonal diversity and use them to analyze several characteristics of the tumor, including its microenvironment, and its impact on muscles. In addition, we improved small animal imaging abdominal oncology protocols (9.4 Tesla MRI) to accurately characterize them for future longitudinal studies, with a special focus, not only on tumor size, but also on intra- and extra tumoral substructural variabilities, reflecting differences in the microenvironment and on muscle characteristics. We also correlated these observations with an immunohistological stratification regarding the expression of several markers (p53, CDX2, HER-2, PD-L1, PD-1, and a-SAM) that are either direct targets of therapies or described markers of therapeutic response. We showed that our PDX models represent a small library of human gastric tumors, with distinctive molecular and histological characteristics, that are precious tools to gain a better understanding of the pathophysiological behavior of GCs and their response to platinum-based therapies.

## Materials and method

### Establishment of patient-derived xenografts (PDX)

PDX were established and treated with cisplatin as described in Venkatasamy et al., 2021; and supplemental methods.

### MRI imaging protocol

Two mice from each established PDX model were imaged using a preclinical 9.4 T MRI (Bruker BioSpin MRI GmbH, BioSpec 94/20, Ettlingen, Germany). The mouse was anaesthetized (Isoflurane®, Abbott GmbH, Wiesbaden, Germany; 2% vaporized in oxygen), and then sacrificed. The MR imaging was performed using a mouse body coil with the mouse carefully placed in the coil in dorsal decubitus and in a prone position. First, an axial RARE T2-weighted sequence was performed, with the following parameters: TR = 24 ms, TE = 4000 ms, average = 1, slice thickness = 0.5 mm, FOV = 28 × 28, image size = 128 × 128, excitation angle = 90°, duration 4–5 min. Then a diffusion-EPI sequence with ADC map (apparent diffusion coefficient), in mm^2^/s displayed as a parametric map and calculated from the diffusion sequence, was performed, using the following parameters: TR = 25.49 ms, TE = 3200 ms, average = 1, flip angle = 90°, slice thickness = 1 mm, FOV = 28 × 28, image size = 128 × 128, diffusion directions = 1 (b0–b200–b500–b1000), duration 4–5 min. All data were analyzed using Paravison® 6.1 software. Paravertebral muscle surface has been measured using the open-access Dicom viewer Horos ®, by a manual segmentation on axial images of a T2-weighted RARE sequence, at the level of the renal hilum, by an experienced radiologist.

### RNA-seq analyses

TO identify the deregulated genes in the mouse microenvironment of the tumor, total RNA was extracted from PDX tumor tissue using a standard TRIZol procedure (TRI Reagent®: TR 118 Molecular Research Center, Cincinnati, OH, USA), according to the manufacturer’s instructions. After extraction and RNA precipitation, supernatants were removed and the RNA pellet was washed with 75% EtOH, centrifuged at 9000×*g* for 5 min at 4 °C, and again, 75% EtOH was added. Then the RNA was resuspended in nuclease-free H2O and quantified using NanoDrop Spectrophotometer (Thermo Scientific, Waltham, MA, USA). RNA sequencing was performed by the GenomEast platform (Strasbourg). RNA-seq library was generated using the Prep Librairie ARN total Ribozero (Ribozero RNA) kit. RNA-seq was performed using a NextSeq 2000 Illumina sequencer, and sequences obtained were selectively aligned on a chimeric genome composed of the mouse genome (mm10) and human genome (hg38) using STAR version 2.5.3a. Quantification step was performed using HTSeq-count version 0.6.1p1, with annotations from Ensembl version 103 (Homo sapiens) and 102 (Mus musculus), and then data were further processed with AltAnalyze version 2. After standard normalization, deregulated genes with log2 fold change > 1.5 and adjusted *p* value < 0.05 were selected, and pathways enrichment analyses were performed using multiple databases (e.g., DAVID, STRING, Reactome, TRAP, Biomarkers).

### Histological analysis

It was performed as described in Venkatasamy et al., 2021 and supplemental methods.

### Immunohistochemistry (IHC)

See supplemental methods.

## Results

### Histological and immunohistological analyses of the GC PDX (GCX) models

**Fig. 1:**
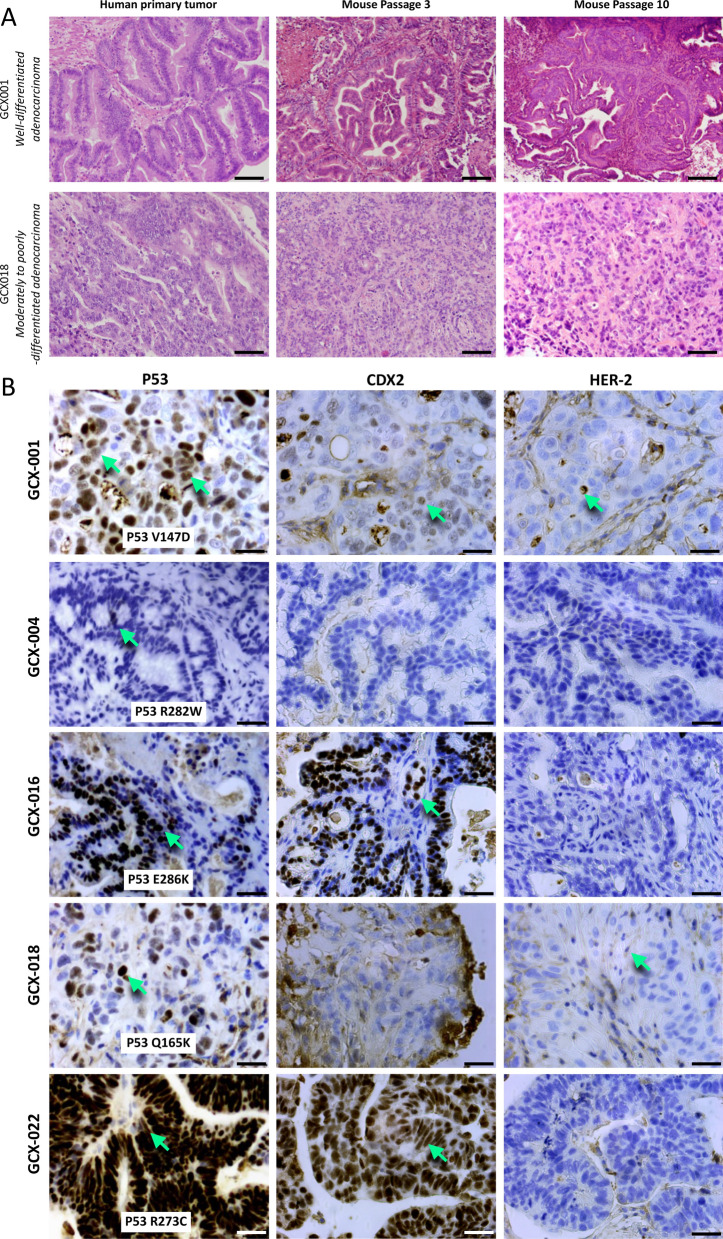
Histological and immunohistology analyses of the GCX models. **A** Immunohistology H&E staining of the primary tumor, mouse passage 3 and 10 of GCX-001, a well-differentiated gastric adenocarcinoma, and GCX-018, a poorly differentiated gastric adenocarcinoma. Magnification × 20; scale bar = 50 µm. **B** Immunohistology analyses of the different GCX models for the expression of P53, CDX2, and HER-2. Green arrows point at clearly positive cells; magnification 40×; scale bar = 20 µm. Insert in the left panel indicates the p53 mutational status of each GCX model

Tumor tissue samples from 24 patients with gastric adenocarcinomas were implanted heterotopically on the flanks of athymic NUDE mice and five different gastric adenocarcinomas tumors were successfully maintained over time (> 10 serial transplantation in mice). These tumors conserved histological structures similar to the parental tissue (Fig. 1A). No lymphomatous transformation [5] of any of these GCX models, even after eight or more passages was observed. The clinical characteristics of the patients from whom the successful grafts were issued are summarized in Table 1. To further characterize the different GCX models, we analyzed them by immunohistochemistry for the expression of p53 (P53), known to be mutated in about 50% of gastric adenocarcinomas [22], CDX2, marker for intestinal gastric cancer subtype [23] and HER2 [25], overexpressed in 15–20% of GC, (Fig. 1B). Except GCX-004, all GCX models showed strong P53 expression (Fig. 1B) suggesting that the cancer cells express a stabilized mutant p53. Sequencing on *TP53* hot spots confirmed that all PDX were expressed at a mutant p53 (Fig. 1B insert). GCX-001, GCX-016, and GCX-022 exhibited strong nuclear CDX2 staining (> 2+), respectively, in 80%, 90%, and 100% of the cells. In contrast, GCX-004 and GCX-018 were negative for CDX2. GCX-001 and GCX-018 showed low level of HER-2 expression (1+) and GCX-004 and GCX-022 were HER-2 negative (Fig. 1B). Interestingly, although the primary human tumor of GCX-016 showed positive HER-2 expression, its mouse GCX counterpart did not (Fig. 1B).

**Table 1:** Clinical characteristics of the patients from whom GCX were established

	Histology	Age	Sex	Preoperative treatment	Surgery type	pTNM	Sample type	Cold ischemic time	Percentage of cells on sample	Time to reach ~ 150 mm^3^	Other
GCX-001	Well-differentiated HER2 + (score 2) gastric adenocarcinoma	76	M	Preoperative chemotherapy EOX protocol (epirubicine + cisplatin + 5-fluoro-uracil)	Distal gastrectomy with Lewis Santy reconstruction	*p*T1b N1 (1/30) M0	Pre-therapeutic biopsy	< 1 h	100%	3 months	
GXC-004	Well-differentiated papillary HER2−gastric adenocarcinoma	64	M	Preoperative chemotherapy using EOX protocol	Total gastrectomy	ypT1aN0 (0/16) M0	Surgical specimen	< 1 h	100%	15 days	No relapse or recurrence after 3 years
GXC-016	Well-differentiated papillary HER2 + (score 2) gastric adenocarcinoma	74	W	Preoperative radio-chemotherapy	Total gastrectomy	pT3N0M0	Pre-therapeutic biopsy	> 1 h	70%	2 months	History of breast cancer. Epidermoid lung cancer diagnosed during follow-up
GXC-018	Moderately differentiated papillary HER2 + (score 1) gastric adenocarcinoma	59	M	Preoperative chemotherapy FLOT protocol (docetaxel + oxaliplatin + 5-fluorouracil + folinic acid)	Total gastrectomy	ypT4aN1 (1/21)M0	Surgical specimen	< 1 h	75%	2 months	No relapse or recurrence after 2 years
GXC-022	Moderately differentiated papillary HER2−gastric adenocarcinoma	66	M	Preoperative chemotherapy FLOT protocol (docetaxel + oxaliplatin + 5-fluorouracil + folinic acid)	Upper pole gastrectomy with Lewis Santy reconstruction	ypT1N0M0	Pre-therapeutic biopsy	< 1 h	< 30%	1 to 2 months	History of lung cancer No relapse or recurrence after 3 years

### Characterization of the tumor microenvironment in GCX models

**Fig. 2:**
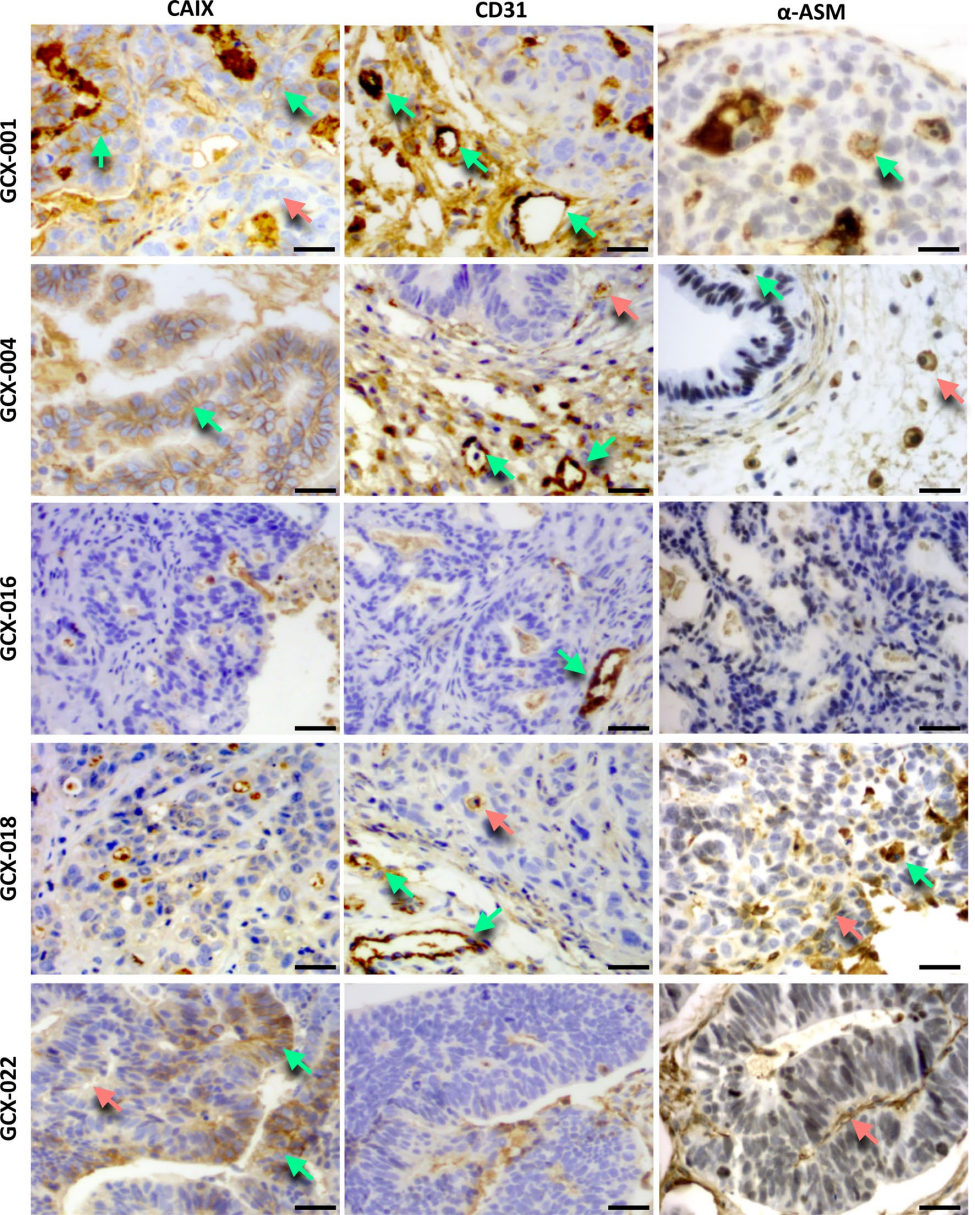
Characterization of the tumor microenvironment. Immunohistology analyses of the different GXC models for the expression of the hypoxic marker CAIX (green arrows pointing at high CAIX labeling, red arrow at low labeling), blood vessel marker CD31 (green arrows pointing at CD31 positive vessel in the stroma, and red arrow at labeling within the cancer cell mass), and the a-smooth muscle protein a-SMA (green arrows pointing at intra-tumor positive staining indicative of macrophages, and red arrow at staining in the stroma). Magnification 40×; scale bar = 20 µm

**Fig. 3:**
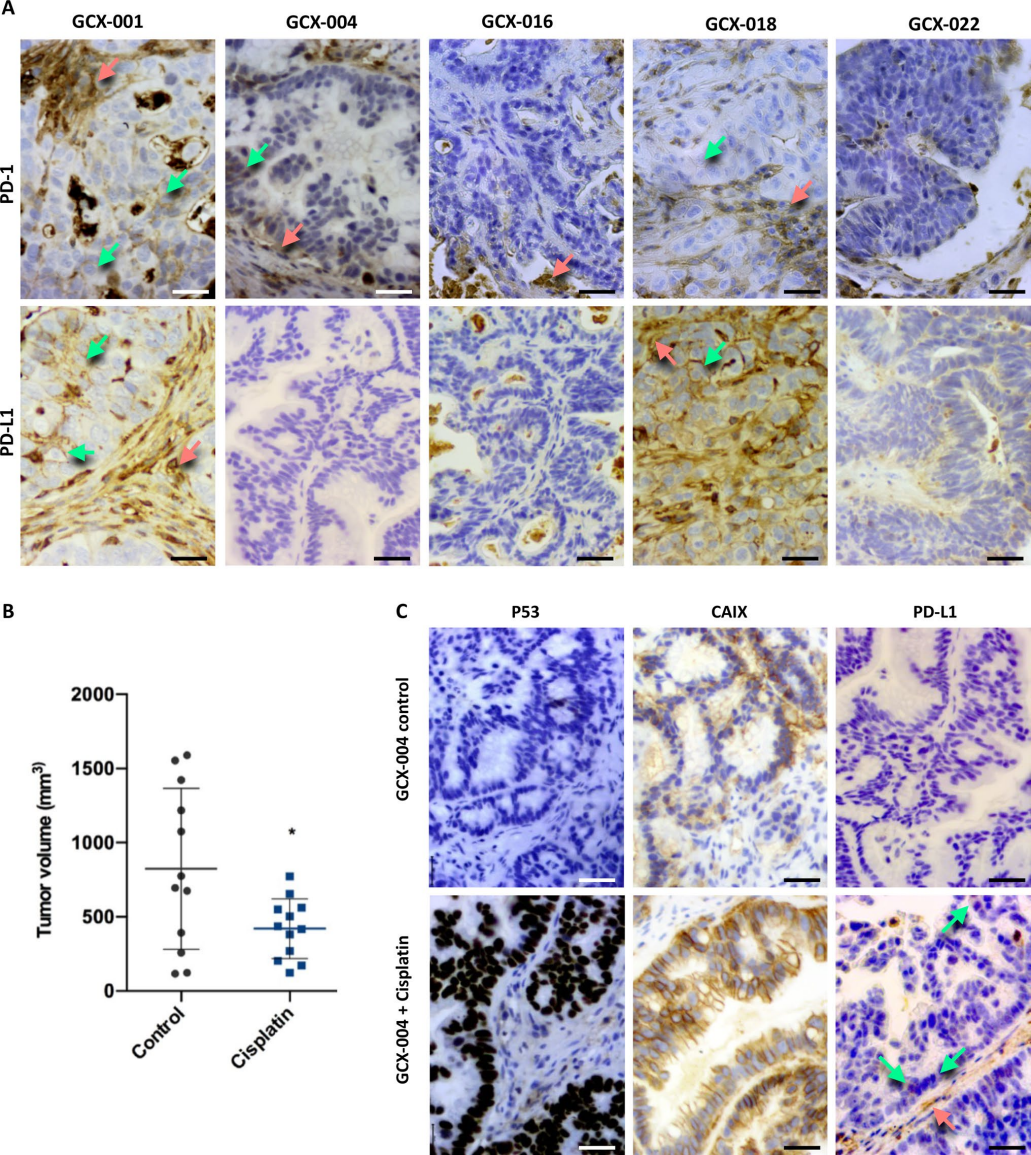
Impact of the chemotherapy on PD-L1/PD-1, p53, and CAIX: **A** immunohistology analyses of the different GCX models for the expression of PD-1 and PD-L1 (green arrows pointing at positive staining in the cancer cell mass and red arrow in the stroma). Magnification 40×. **B** Nude mice bearing 50 mm^3^ GCX-004 tumors were treated with cisplatin, and tumor volume was measured after 23 days. **C** Immunohistochemical analyses of p53, CAIX, and PD-L1 (green arrows show positive staining in the cancer cells and red arrow in the stroma), showing a clear induction of their expression after cisplatin treatment. Magnification 40×; scale bar = 20 µm

To characterize the tumor microenvironment, we first analyzed the presence of hypoxic regions within the tumor, using carbonic anhydrase as a marker (CAIX, Fig. 2). Immunohistochemical evaluation of CAIX staining showed a strong membranous staining (2+/3+ green arrows) in 100% of the cells of GCX-004, whereas GCX-001 and GCX-022 showed a heterogenous staining with cell expressing high levels of CAIX, 60–70% in GCX-001 and 20% in GCX-022 (green arrows), respectively, and cells showing low expression (red arrows). No CAIX membranous staining was observed in GCX-016 and GCX-018. We next evaluated the tumor vascularization by staining for CD31 expression (Fig. 2, center panel, green arrow pointing to vessels in the stroma and red arrow within the cancer cell mass). GCX-001, GCX-004, GCX-018, and GCX-022 presented intra-tumoral neovascularization, showing small vessels between the glands and around the tumors. In addition, expression of α-smooth muscle actin (α-SMA, Fig. 2, right panel) was detected within the cancer cell mass (GXC-001, GCX-004, and GCX-0018, green arrows) or/and in the stroma (GCX-004, GCX-016, and GCX-022 tumors, red arrows) of tumors, indicating the presence of cancer-activated fibroblasts. We also examined the expression of the immune checkpoint inhibitor ligand PD-L1 which interacts with the PD1 receptor expressed on T-cells inhibiting, thereby their activation and elimination of the cancer cells by the immune system. The immunohistochemistry analyses showed that GCX-001 and GCX-018 presented a clear positive PD-L1 and PD-1 membrane staining of cancer cells (Fig. 3A, green arrows) and cells in the tumor microenvironment (red arrows). The other models were either negative or showed low levels of PD-L1/PD-1 expression (GCX-004 = PD-L1 negative/PD-1 < 5%, GCX-016 = PD-L1 negative/PD-1 negative, GCX-022 = PD-L1 < 10%/PD-1 < 5%).

### Impact of the chemotherapy on PD-L1/PD-1, p53, and CAIX in GCX models

To investigate the impact of chemotherapy on the tumor microenvironment, we treated GCX-004 mice with cisplatin and analyzed GCX-004 tumors for the expression of P53, CAIX, and PD-L1 by immunohistochemistry (Fig. 3A). Importantly, cisplatin significantly reduced tumor growth in the GXC-004 mouse model (Fig. 3B). The expression of p53 and the hypoxic marker CAIX (Fig. 3C) was strongly increased by the cisplatin treatment. Cisplatin also slightly increased the expression of PD-L1 both in tumor cells and in the stroma (Fig. 3C green and red arrows, respectively). This shows that cisplatin not only has an impact on gene expression within tumor cells but also on the tumor microenvironment.

### Radiological characterization of GCX models

**Fig. 4:**
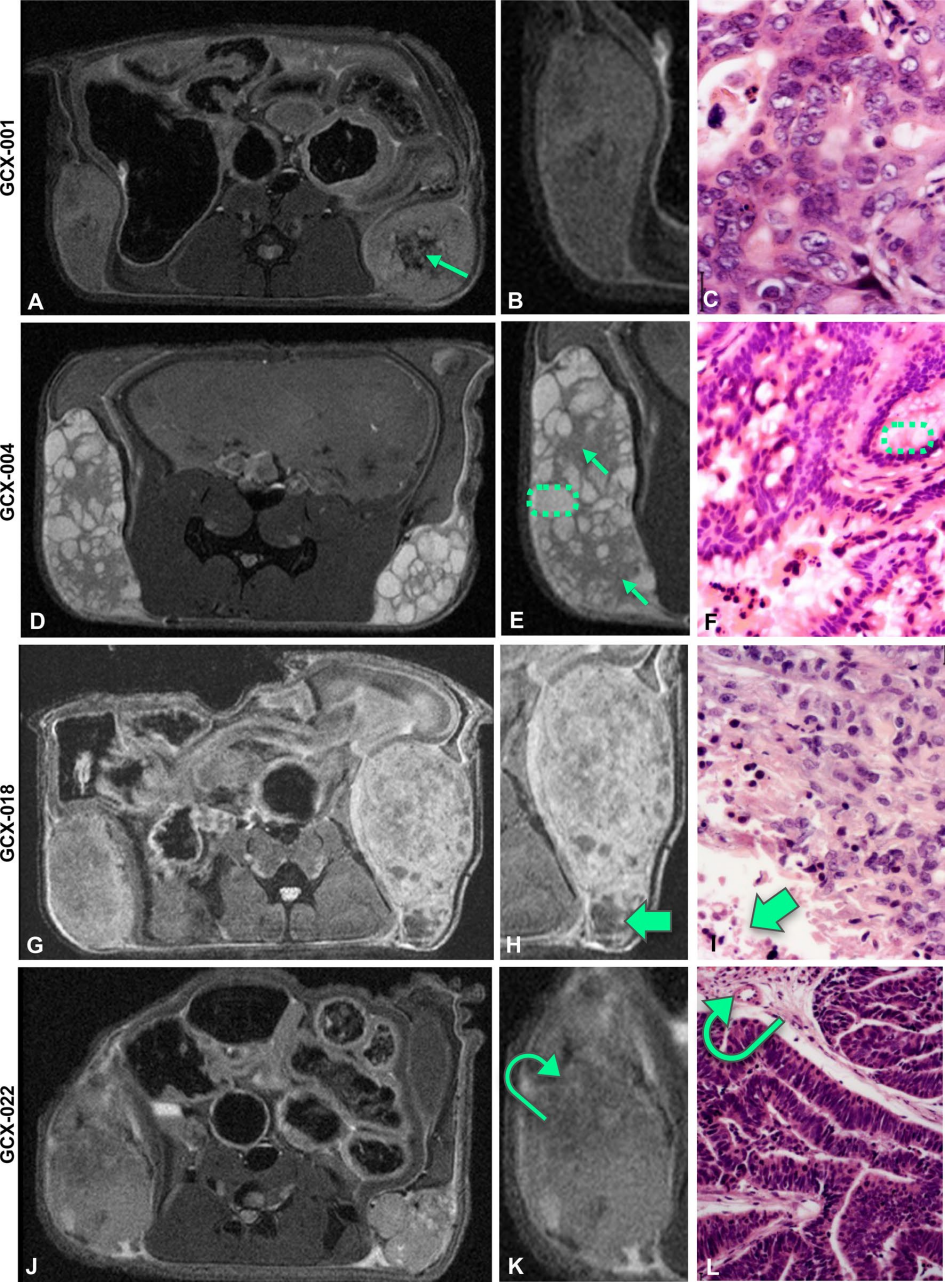
Characterization of tumors’ substructures using a 9.4 T MRI. **A**, **D**, **G**, **J** T2-RARE images of the indicated GCX model, **B**, **E**, **H**, **K** close up of the respective GCX tumor, and **C**, **F**, **I**, **L** H&E staining of the respective GCX tumor. **A** The green arrow indicates the localization of necrosis within the tumor. **E**, **F** Green arrows point to hypointense fibrous stalks and green dash line circles to clumps of edematous papillae. **G**–**I** Tumors appear more heterogenous, with multiple foci of necrosis (thick green arrows). **K**, **L** Curved green arrow points to neo-vasculature structures, predominantly peripheral, visible as linear hypointense structures on T2-weighted images

**Fig. 5:**
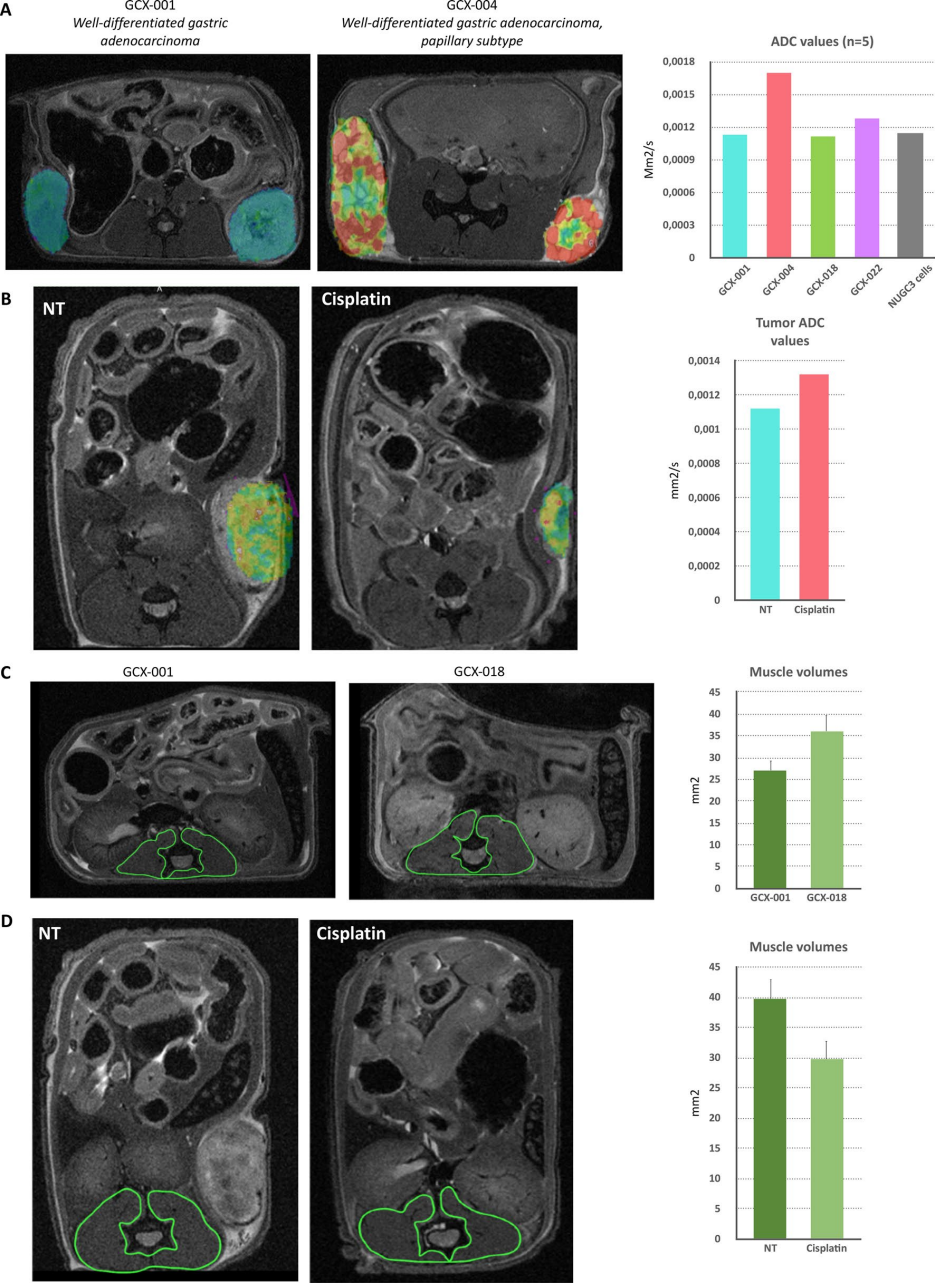
Radiological characterization revealed differences in tumor substructure and muscular atrophy between different GCX models and after cisplatin treatment. **A** Fusion image obtained combining a T2-RARE image and its corresponding ADC color map of GCX-001, a well-differentiated gastric adenocarcinoma, and GCX-004, a well-differentiated gastric adenocarcinoma of the papillary subtype. Hyperintense clumps of edematous papillae are identified by the red to yellow color on the ADC map. Graph to the rights indicates the respective ADC values for each GCX model. **B** ADC color map and measurement of GCX-001 treated or not with cisplatin. **C**, **D** Comparison of muscle volumes, between two GCX models and GCX-001 treated or not with cisplatin, by T2-RARE imaging with manual delineation (green) of the paravertebral muscles. Graphs to the right show the corresponding values of the volume of the paravertebral muscles, and bars indicate average with standard deviations

PDX models are valuable tools to perform longitudinal pre-clinical studies for drug testing by assessing the tumor size and structure, but also to evaluate potential side effects on healthy tissue (e.g., muscle). Hence, we used magnetic resonance imaging (MRI) as it can provide in vivo direct access to information about the tumor size, morphology, substructure, and to potential treatment-related changes. To do so, we have imaged four GCX models using a 9.4 Tesla MRI (Fig. 4, left panel) with a dedicated mouse coil, using T2-weighted and diffusion-weighted sequences, which we specially improved for murine abdominal oncology. The appearance and signal intensity on the T2-weighted images varied between the different GXC-models (Fig. 4A and B), according to the tumor tissue characteristics and its fluid content. For instance, GCX-001, a well-differentiated gastric adenocarcinoma, appeared homogenous (Fig. 4A and B), presenting an intermediate signal on T2-weighted images and well-delineated peripheral margins. The left flank tumor (Fig. 4A, middle panel) appeared slightly more heterogenous than the right flank tumor, with central areas of necrosis (green arrow). As expected, GCX-004 model, a well-differentiated intestinal gastric adenocarcinoma, (Fig. 4D–F) presented the classical appearance of a papillary tumor on MRI: lobulated with hypointense fibrous stalk (appearing grey on the MRI images, green arrows, Fig. 4E) supporting clumps of hyperintense fluid-like (i.e., bright signal intensity similar to that of fluid, encircled by green dash lines on both MRI and pathology images; Fig. 4E, F) edematous papillae on T2‐weighted images. The GCX-018 model (Fig. 4G–I), a moderately to poorly differentiated gastric adenocarcinoma presented with a heterogenous signal, due to large foci of necrosis visible also on histology (Fig. 4H, I, green arrow). The GCX-022 model (Fig. 4J–L) showed neo-vasculature, especially in the periphery of the tumor, presenting as linear hypointense structures on T2-weighted images also observed on histology (Fig. 4K, L curved arrow). To get further insights into the histological architecture of the GCX tumors, we used diffusion-weighted imaging which relies on the detection of the random motion of free water molecules within the tissues. Restriction of water diffusion can then be quantified by apparent diffusion coefficient (ADC) [25] giving information about the histological architecture of the tissue. For instance, a hyperintense (i.e., bright) signal on diffusion-weighted images together with low ADC values is indicative of high cellularity, in favor of a malignant tumor. In addition, a hyperintense signal on diffusion-weighted images with higher ADC values can be related to due to higher fluid content within the tumors, causing increased diffusion of the water molecules within the tumor. All PDX tumors showed restricted diffusion, related to high cellularity (Fig. 5A: their respective ADC values were 0.00113 mm^2^/s for GCX-001, 0.00128 mm^2^/s for GCX-022, and 0.00112 mm^2^/s for GCX-018). As expected, the GCX-004 PDX, which showed higher signal on T2-weighted images, also presented with slightly higher ADC value (0.00170 mm^2^/s) than the other GCX models, probably due to the fluid-like content of its papillae (Fig. 5A). Importantly, although cisplatin decreased tumor size, ADC values were clearly increased (Fig. 5B).

As we previously observed that muscular atrophy is associated with gastric cancer both before and after chemotherapy treatment [14, 15, 26], we decided to assess the muscle status in the GCX models. For this, we investigated the paravertebral muscles of the PDX mice using the same measurement method performed on cancer patients to diagnose muscle atrophy on abdominal images of CT or PET–CT [27]. Interestingly, our five GCX models presented differences in terms of paravertebral muscle surface. The average paravertebral muscle surface (measured at the level of the renal hilum) was 34 ± 6 mm^2^, with up to 28% variation between models. For instance, the paravertebral muscle surface values were higher for GXC-018 (39 mm^2^ Fig. 5C) compared to GCX-001 (27 mm^2^ Fig. 5C). Hence, depending on the characteristics of the initial tumor and in absence of treatment, muscular atrophy can occur in mice, mimicking what is observed for gastric cancer patients. Importantly, like in humans [13, 27], cisplatin impacted negatively on muscle mass in mice (Fig. 5D), reducing its volume by 22% (39 mm^2^ vs 30.5 mm^2^).

### Cisplatin induces gene expression changes in GCX models

**Fig. 6:**
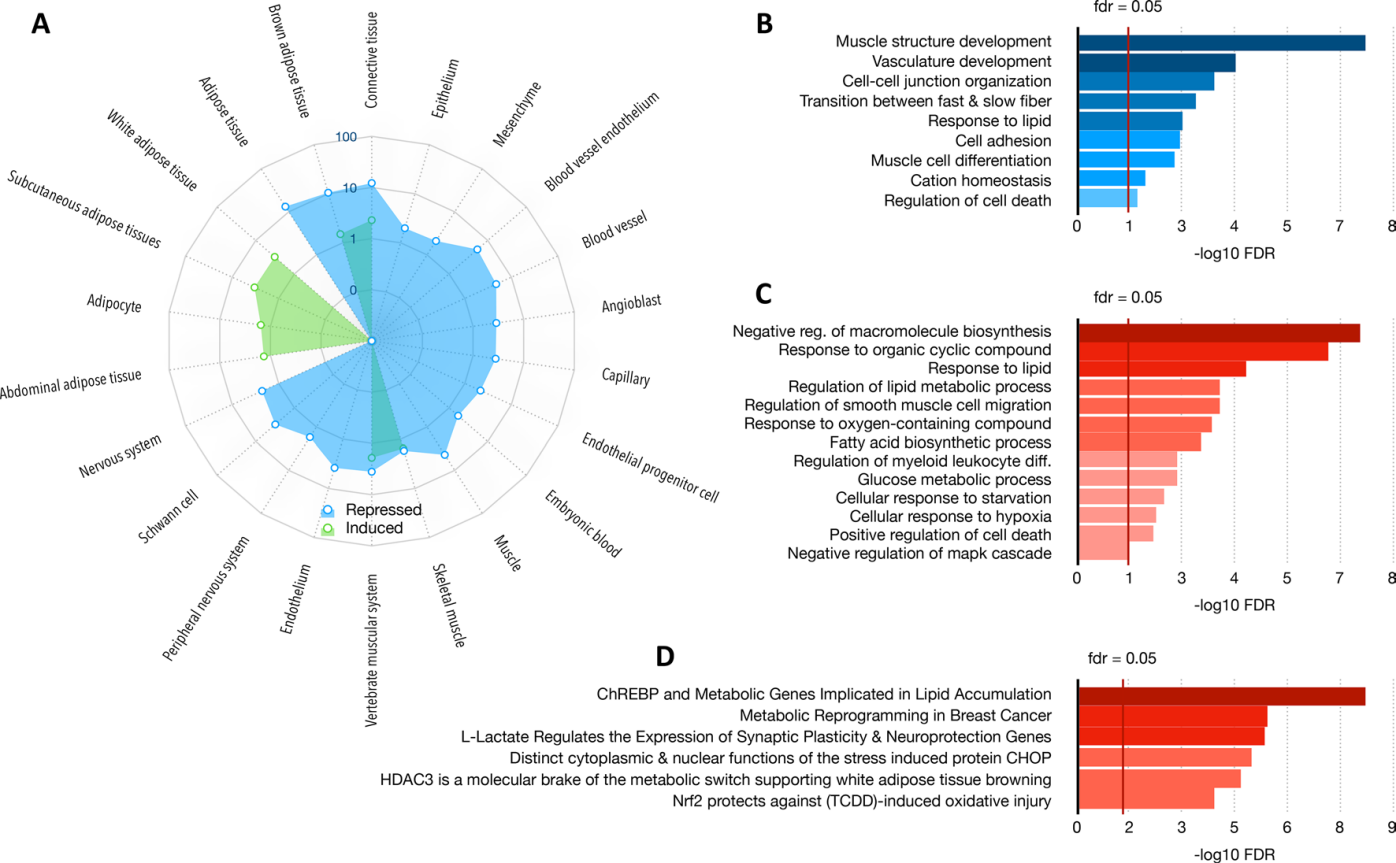
Impact of cisplatin on gene expression in the tumor microenvironment. RNA-seq experiment was performed on the murine cells of the tumor (stroma). Comparative gene expression, clustering and pathways analyses identified deregulated genes that segregated within defining markers for given cell types and cellular processes. Gene used for clustering and pathways analyses were selected based on fold change (> 1.3) and *p* value (< 0.05). **A** clustering analysis using STRING identified changes in cell type signatures after cisplatin treatment, with decreases in muscle-like, neuronal-like and capillary-like cell types. In contrast, an increase in adipocyte-like cell type is observed. **B** Biological processes identified as repressed after cisplatin treatment. **C** Biological processes identified as upregulated after cisplatin treatment. **D** Transcriptional deregulations induced by cisplatin

High-resolution imaging and histological/immunohistochemistry analyses showed that cisplatin impacts on the tumor ecosystem by affecting the muscle mass and the tumor microenvironment. To confirm these differences, we analyzed gene expression changes in human gastric tumors of GCX-004 mice which have been treated or not with cisplatin by RNA-seq. The sequences obtained were specifically aligned on the mouse genome, allowing us to analyze the mouse microenvironment rather than the human cancer cells. After normalization, deregulated genes were selected according to an absolute value of a log2 fold change > 1.5 and an adjusted *p* value < 0.05. The list of resulting genes was then used to identify altered pathways and specific makers in each experimental condition using the webtool STRING. Clustering analysis identified genes corresponding to given tissues that were either induced, such as subcutaneous adipose tissues and adipocytes, while others were repressed, such as blood vessel endothelium, muscle or nervous system (Fig. 6A). Similarly, genes associated with specific biological processes were also downregulated, such as those involved in muscle structure development, cell adhesion or vasculature development (Fig. 6B). Inversely, genes indicative of a response to cyclic organic compounds or oxygen species or hypoxia, and of glucose or lipid metabolism were induced (Fig. 6C). The metabolic and cellular/tissue changes in the tumor microenvironment indicated by the gene expression patterns are also supported by genes segregating within signatures indicative of the activity of relevant proteins/transcription factors, such as ChREBP activity for lipid metabolism or NRF2 in response for oxidative injury (Fig. 6D). Taken together, the transcriptomic analyses confirmed the initial observations that cisplatin impact on the mouse microenvironment and likely distant tissues, such as the muscles.

## Discussion

In preclinical studies, the effect of a treatment on a tumor is usually accessed through the measure of its volume and its speed of growth, using cell line-derived or patient-derived mouse models. PDX models have been shown to remain stable across generations retaining the major traits of their originating tumors [5, 19, 23–25]. This is what we observed in our gastric cancer PDX model, with tumors maintaining the same histology and heterogeneous expression of molecular markers (such as p53, CDX2 or PD-L1/PD-1) across generations. In particular, PDX models allow repeated measures and time-course follow-up of tumor progression, in response to various conditions (e.g., anti-cancer drugs, genetic modifications…), as well as an analysis of the evolution of intra-tumoral clonal variation [16–21]. In our case, using a MRI-based approach, we observed a chemotherapy-induced impact on tumor growth that depended on tumor type. In addition, changes in muscle mass were observed upon treatment using a scanner-based approach. After cisplatin chemotherapy, GCX004 tumors showed histological changes (stroma predominance and decreased tumor burden) as well as protein expression changes, especially a very strong increase in nuclear p53 staining. Similarly, cisplatin induced hypoxia within tumor cells. However, and interestingly, our RNA-seq analyses demonstrate that platinum-based chemotherapy also induces gene expression changes in the tumor microenvironment indicative of muscle cells, vascular endothelial cells, and lipid metabolism changes. These changes in the tumor microenvironment were further illustrated and supported by high-resolution imaging, such as increased ADC measurements indicating of cell death [28]. Characterization of the tumor microenvironment showed that PDX tumors behaved like human GC. Notably, 40% of PDX strongly expressed PD-L1 (> 50% positive membrane staining), which agrees with human observations where PD-L1 expression was noted in around 30% of gastric cancer tumor cells [9]. Currently, GC patients are only stratified according to their HER2 and PD-L1 expression. However, how standard chemotherapeutic treatments impact on their expression and GC tumor microenvironment and its immunogenicity of GC cells is still largely unknown. In this regard, our results clearly show that cisplatin upregulates the expression of PD-L1 in GCX004 cells suggesting that also for those patients showing PD-L1 negative or low tumors, a co-treatment with nivolumab or pembrolizumab (for the treatment of GC FDA-approved anti-PD1/PD-L1 anti-bodies) might further improve the therapeutic outcome. This is a crucial information when considering combining immunotherapy with other types of therapies. However, in contrast to published date, we did not see a correlation between p53 expression and PD-L1/PD-1 levels, in contrast to a recent publication [29].

Another important part of the tumor ecosystem is its dynamic interactions with more distant tissues, in particular the muscle, which is affected by a cancer-related atrophy. This is particularly the case in GC, for which 6 to 13% of patients have sarcopenia at diagnosis and 60% develop it upon chemotherapy [13]. In all cancers, cachexia and sarcopenia probably have complex origins, partially related to the tumor itself and partially induced, or worsened, by treatments. Our study represents the first work that investigates the impact of human gastric tumors and/or anti-cancer treatment on muscles, using PDX models. Our results show that both, the tumor itself as well as that platinum-based anti-cancer drugs like cisplatin can inflict sarcopenia. In the case of GC, some clinicians explain the muscle atrophy by the fact that patients cannot eat normally, as the gastric tumor alters the capacity of correctly ingesting and digesting food. However, we detected associated cachexia even with GC tumors growing subcutaneously on the flanks of the mice, which does not lead to any compression of the stomach or digestive organs. This strongly suggests that factors secreted by the tumor (e.g., IL6) or the interaction with the tumor environment contribute to the development of cancer cachexia. As indicated in the literature for other cancers, tumor secreted molecules such as IL6 [29]. Considering this, precisely identifying those factors, which are secreted by the gastric tumor cells or its microenvironment, contributing to the development of cachexia might open up new perspectives for development therapeutic strategies.

Taken together, the five patient-derived xenografts enabled us to obtain a quite complete overview of intestinal-type gastric cancer tumors, reproducing well the heterogeneity and the complexity of human tumors, showing differences in terms of tumor structure, histology, P53/PD-L1 status, and muscle atrophy, which makes them even more useful in preclinical research, for the development of novel drugs and targeted therapies. Importantly, our results also highlight the complexity of patients’ response to platinum-based anti-cancer drugs like cisplatin, which not only impact the tumor growth but also the tumor ecosystem (i.e., close environment and remote tissues) and that this should be considered for the development of combinatory or new therapeutic protocols.

## Supplementary Information

Below is the link to the electronic supplementary material.

Supplementary file1 (DOCX 16 KB)

## Data Availability

The data in this study are available from the author for correspondence upon reasonable request.
