# Supplementary material for: Morpho-functional analysis of patient-derived xenografts reveals differential impact of gastric cancer and chemotherapy on the tumor ecosystem, affecting immune check point, metabolism, and sarcopenia
Source: Gastric Cancer. 2022 Dec 19;26(2):220–33. doi: 10.1007/s10120-022-01359-w (PMC9950243; doi:10.1007/s10120-022-01359-w)
Supplement: Supplementary file 1 — Supplementary file1 (DOCX 16 KB) [file 10120_2022_1359_MOESM1_ESM.docx]

**Mouse high-resolution imaging:**

Animals underwent an abdominal micro-CT at day 0 – day 3 – day 5 (Institut Pluridisciplinaire Hubert Curien). We also performed, *ex vivo* magnetic resonance imaging using a preclinical 9.4T MRI (Bruker BioSpin MRI GmbH, BioSpec 94/20, Ettlingen, Germany) and a mouse body-coil. The mice were placed in the coil in dorsal decubitus in a prone position (head first). We performed an axial RARE T2-weighted sequence, with the following parameters: TR = 24ms, TE= 4000ms, average = 1, slice thickness = 0.5mm, FOV = 28 x 28, image size = 128 x 128, excitation angle = 90°, duration 4-5 minutes. Then, we performed a diffusion-EPI sequence with ADC map, using the following parameters: TR = 25.49 ms, TE= 3200 ms, average= 1, flip angle = 90°, slice thickness = 1mm, FOV = 28 x 28, image size = 128 x 128, diffusion directions = 1 (b0 – b200 – b 500 – b1000), duration 4-5 minutes. We performed a T1-weighted in / out of phase sequence, using the following parameters: TR = 300ms, TE= 1,43/1,79ms, average= 2, flip angle = 30°, slice thickness = 1mm, FOV = 28, image size = 140x140, duration 1m 24 sec. We also performed a T1-weighted sequence (with and without FS), using the following parameters: TR = 300ms, TE= 3ms, average= 2, flip angle = 30°, slice thickness = 1mm, FOV = 28, image size = 140x140, duration 1min 3s.

Surface measurements of paravertebral muscles were measured on an axial plane at the level of the renal hilum and signal intensity were measured on the T1-weighted sequences using Region of Interest (ROI) with the free DICOM viewer Horos ^TM^ and Paravison® 6.1 software.

**Immunohistochemistry (IHC):**

IHC analyses were performed on deparaffinized histological tumor slices (5 µm). Unmasking was carried out with NaCitrate in the microwave oven (800 W, 10 min). After washing once with distilled water and once with 1xPBS, the sections were permeabilized with 1xPBS-0.1% Triton for 5 min and the nonspecific sites blocked in PBS-0.1% Triton-5% Normal Goat Serum (NGS) during 60 min. Subsequently, primary antibodies to P53 DO1 (BP53-12, Zytomed), P63 (4A4, Ventana 790-4509), PD-L1 (22-C3, Dako M3653), PD-1 (NAT105, Abcam ab52587), CDX2 (EPR2764Y, diagomic's Zytomed RBK012-05), α-smooth muscle actin (1A4, Dako M0851), CD31 (Microm MS Ac-0083), CAIX (Abcam Ab15086), and HER-2 (4B5, Ventana790-4493) were diluted in 1xPBS-0.1% Triton then added to slides overnight at 4°C. After 3 washes with 1xPBS-0.1% Triton, slides were incubated with the secondary antibody (diluted 1:1000 in 1xPBS/5% NGS) specific for the primary antibody for 1 h at room temperature. Slides were mounted with FluorSafe Reagent Mounting Medium (Calbiochem®) and allowed to dry overnight before observation using a fluorescence microscope (Axio Imager 2 - ZEISS). The staining was scored as % of cells showing a positive staining (nuclear for P53, CDX2, P63, membrane for PD-L1 and HER-2, membrane and cytoplasmic for α-actin-smooth-muscle).

**Establishment of Patient-Derived xenografts in nude mice (PDX):**

Use of human tissues was approved by the Ethics board. Patients gave their written consent. All protocols adhered to the Declaration of Helsinki. The prospective clinical and histological data included: age, sex, weight, height, type and histology (Lauren’s classification), TNM stage, treatment, *p*TNM after gastrectomy, the occurrence of post-operative complications and the patients’ clinical evolution. The primary tumor fragment used for implantation was issued from either gastric cancer biopsies (2x2 mm) or tumor fragments (4x4 mm) of surgical specimens of gastric adenocarcinomas gathered under sterile conditions, with limited cold ischemic time (<25 min). All animal experiments were approved by the Institutional Animal Care and Use Committee (APAFIS#8320). The mice were cared for according to the Institutional Guidelines for Animal Care. Six weeks-old athymic female NUDE mice were acclimated for two weeks, provided with unlimited access to sterilized food and water *ad libitum,* and housed with 12h day/night cycle. The primary tumor fragments/biopsies were implanted on the flanks of anaesthetized (Isoflurane®, Abbott GmbH, Wiesbaden, Germany; 2% vaporized in oxygen) NUDE mice, with close monitoring of their growth (1-3 times/week by caliper). When the implanted tumor reached 500mm^3^, the engraftment was considered successful, the mouse was anaesthetized, and the tumor harvested for serial transplantation into mice. To establish the PDX models, tumor implantations were performed in different generations of mice (passage 1 to passage 10), to verify the reproducibility of the implantation, but also the conservation of the tumor major characteristics (architecture, histology, genetics…) between the serial passages.

**Treatment protocol and tumor measurement:**

For our experiment, tumor fragments (~3mm^3^) from *GCX-004* (passage 8) were implanted subcutaneously on both flanks of 12 NUDE mice, and the mice were split into two groups. Once the tumors reached 50mm^3^, we treated one group (*n*=6) with cisplatin (5mg/kg body weight and the control group (n=6) with DMSO-PBS-cremofor. Tumor volume was measured after 23 days by caliper. For caliper measurements, the thickness of the tumor was extrapolated from the width measure. The volume was calculated as V= (length x width) 2/2
